# Absence of genotoxic effects of the chalcone (*E*)-1-(2-hydroxyphenyl)-3-(4-methylphenyl)-prop-2-en-1-one) and its potential chemoprevention against DNA damage using *in vitro* and *in vivo* assays

**DOI:** 10.1371/journal.pone.0171224

**Published:** 2017-02-16

**Authors:** Débora Cristina da Silva Lima, Camila Regina do Vale, Jefferson Hollanda Véras, Aline Bernardes, Caridad Noda Pérez, Lee Chen-Chen

**Affiliations:** 1 Department of Genetics, Institute of Biological Sciences, Federal University of Goiás, Goiânia, GO, Brazil; 2 Institute of Chemistry, Federal University of Goiás, Goiânia, GO, Brazil; IIT Research Institute, UNITED STATES

## Abstract

The chalcone (*E*)-1-(2-hydroxyphenyl)-3-(4-methylphenyl)-prop-2-en-1-one), or 2HMC, displays antileishmanial, antimalarial, and antioxidant activities. The aim of this study was to investigate the cytotoxic, genotoxic, mutagenic, and protective effects of 2HMC using the Ames mutagenicity test, the mouse bone marrow micronucleus test, and the comet assay in mice. In the assessment using the Ames test, 2HMC did not increase the number of His^+^ revertants in *Salmonella typhimurium* strains, demonstrating lack of mutagenicity. 2HMC showed no significant increase in micronucleated polychromatic erythrocyte frequency (MNPCE) in the micronucleus test, or in DNA strand breaks using the comet assay, evidencing absence of genotoxicity. Regarding cytotoxicity, 2HMC exhibited moderate cytotoxicity in mouse bone marrow cells by micronucleus test. 2HMC showed antimutagenic action in co-administration with the positive controls, sodium azide (SA) and 4-nitroquinoline-1-oxide (4NQO), in the Ames test. Co-administered and mainly pre-administered with cyclophosphamide (CPA), 2HMC caused a decrease in the frequency of MNPCE using the micronucleus test and in DNA strand breaks using the comet assay. Thus, 2HMC exhibited antimutagenic and antigenotoxic effects, displaying a DNA-protective effect against CPA, SA, and 4NQO carcinogens. In conclusion, 2HMC presented antimutagenic, antigenotoxic and moderate cytotoxic effects; therefore it is a promising molecule for cancer prevention.

## Introduction

Chalcones (1,3-diphenyl-2-propen-1-one) are naturally occurring aromatic ketones, consisting of an *α*,*β*-unsaturated carbonyl system joining two aryl rings [1]. Chalcone is a class of open-chain flavonoids, abundantly found in plants of the families Leguminosae, Compositae, and Moraceae [2], and present in fruits, vegetables, grains, roots, flowers, tea, and wine, products regularly used for human consumption [3].

Significant progress has been made over the past few years in the research of chalcones not only biosynthesized by plants, but also synthetically prepared. 2’-hydroxychalcones, known for their effectiveness in gene expression and enzyme activity [4,5], are the most important intermediates in the synthesis of flavonoids and flavones [6,7]. This group of chalcones also exhibits remarkable anticancer [8] and antioxidant [9] activities, besides having potential as a neuroprotective and analgesic agent, as well as a cognitive enhancer [10]. So far, a large variety of 2’-hydroxychalcone compounds have been synthesized and studied aiming to obtain promising therapeutic agents.

The chalcone (*E*)-1-(2-hydroxyphenyl)-3-(4-methylphenyl)-prop-2-en-1-one), or 2HMC, has already been shown to have potential pharmacological applications as an antileishmanial and antimalarial agent [11,12]. It has also been considered a promising molecule for anticancer therapy, since it has been proven to have antiproliferative activity against MCF-7 human breast cancer cells [13], to inhibit cathepsin B (protease overexpressed in human primary tumors) [14], and to inhibit the endogenous tumor promoter prostaglandin-E2 (PGE2) in the murine macrophage cell line RAW 264.7 [15]. Given the pharmacological and therapeutic potential of 2HMC, this compound has attracted great interest. Therefore, it is of great importance to investigate its mutagenic, genotoxic, and protective effects against DNA damage.

Several *in vitro* and *in vivo* tests have been combined to evaluate the mutagenic/genotoxic activity of numerous compounds. The combination of these tests has been generally employed due to the possibility of using various target organs in the same animal as well as the feasibility of evaluating different genetic endpoints [16]. Among the most used tests in genetic toxicology, the Ames mutagenicity test (*in vitro*), the mouse bone marrow micronucleus test, and the comet assay (*in vivo*) stand out.

The Ames mutagenicity test uses several different tester strains of *Salmonella typhimurium* to measure two classes of gene mutation, namely base pair substitution and frameshifts [17]. The micronucleus test is widely used to detect clastogenicity and aneugenicity of chemicals [18]. Another recommended test is the comet assay, also called the single cell gel electrophoresis assay, which can determine DNA strand breaks after *in vivo* exposure to the test compound [19].

Compounds that prevent mutagenic or genotoxic events affecting DNA can act by different mechanisms. Some compounds reduce the mutagenic or genotoxic effect by directly interacting with the mutagens or by blocking their effects through the inhibition of their metabolic activation or the enhancement of their detoxifying enzymes (usually detected in pre-treatment or co-treatment). Other substances act after the damage has already taken place by promoting DNA repair (which can be detected in post-treatment), increasing the fidelity of DNA replication, inhibiting error prone replication, or suppressing the growth and replication of cells with damaged DNA [20]. Thus, depending on the type of treatment, it is possible to suggest different mechanisms of action to a test compound.

Considering the promising capacity of 2HMC in cancer therapy and its several pharmacological activities, the aim of the present study was to evaluate the mutagenic/genotoxic and antimutagenic/antigenotoxic effects of this compound using the Ames mutagenicity test in *S*. *typhimurium* strains, the micronucleus test, and the comet assay in mice.

## Materials and methods

### Chemicals

All commercially available reagents were purchased from Sigma-Aldrich (St. Louis, MO, USA). ^1^H NMR (Proton Nuclear Magnetic Resonance) and ^13^C NMR (Carbon-13 Nuclear Magnetic Resonance) spectra were recorded on a Bruker Avance III (500 MHz) spectrometer (Bruker Optik GmbH, Ettlingen, Germany). Infrared (IR) spectra were performed on a Bomem M102 spectrometer (Bomem Inc., Vanier, Quebec, Canada). The melting point was measured using a melting point apparatus (Karl Kolb GmbH & Co., Dreieich, Germany). The purity of the compound was determined by high performance liquid chromatography (HPLC) using the Waters Alliance–2695 apparatus, XTerra C18 (5 μm, 4.6 mm × 150 mm) column, and Waters 2998 Photodiode Array (PAD) detector at 330 nm for quantification of chalcone 2HMC. The injector was programmed to inject a volume of 10 μL. The mobile phase was CH_3_CN:aqueous buffer containing 0.1% trichloroacetic acid (55:45).

### Synthesis of chalcone 2HMC (1)

**Fig 1:**
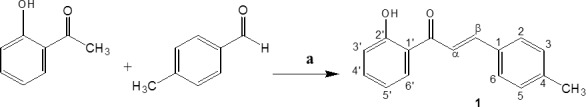
Synthetic route of chalcone 2HMC (1). Reagents and conditions: (a) 50% NaOH (w/w), EtOH, 12 h (room temperature).

The chalcone (*E*)-1-(2-hydroxyphenyl)-3-(4-methylphenyl)-prop-2-en-1-one), or 2HMC, was synthesized in the Institute of Chemistry, at Federal University of Goiás, Goiânia, GO, Brazil. The synthesis of the chalcone is outlined in Fig 1.

Chalcone 2HMC was synthesized by Claisen-Schmidt condensation as previously described in the literature [11,21] with small modifications. Equimolar portions of *o*-hydroxyacetophenone (1.0 mmol) and *p*-methylbenzaldehyde (1.0 mmol) were added to 30 mL 50% NaOH ethanol solution (w/w). The reaction mixture was stirred at room temperature for 12 h, poured into cold water (6 mL), and acidified with 10% hydrochloric acid (w/v) to pH 3, forming a yellow solid compound, which was crystallized with chloroform. The melting point, IR (Infrared), ^13^C NMR, and ¹H NMR are in agreement with previous reports [11,14].

### (*E*)-1-(2-hydroxyphenyl)-3-(4-methylphenyl)-prop-2-en-1-one

Yellow crystals yielded 67%. Melting point: 112–115°C. IR (KBr) cm^-1^: 3436 (ν OH), 1647 (ν CO), 1564 [ν C = C (sp^2^)]. ¹H NMR (Hz–DMSO-d^6^) *δ*: 12.61 (s; 1H; OH), 8.26 (dd; 1H; *J* = 8.47; *J* = 1.75; H6’), 7.99 (d; 1H; *J* = 15.47; H*β*), 7.83 (d; 1H; *J* = 15.47; H*α*), 7.81 (d; 2H; *J* = 8.17; H2, H6), 7.57 (ddd; 1H; *J* = 8.17; *J* = 7.30; *J* = 1.75; H4’), 7.29 (d; 2H; *J* = 8.17; H3, H5), 7.01 (m; 2H; H5’, H3’). ^13^C NMR (DMSO-d^6^) *δ*: 192.93 (CO), 161.99 (C2’), 145.07 (Cβ), 141.30 (C4), 136.36 (C4’), 131.78 (C1), 129.69 (C6’), 129.30 (C2 and C6), 120.73 (C1’), 120.60 (C5’), 119.23 (Cα), 117.80 (C3’), 21.18 (CH_3_). Puritiy of 98.86% in HPLC [retention time = 8.604 min; CH_3_CN: Cl_3_CCOOH buffer (55:45)]. A coupling constant of 15.47 Hz for the vinyl H-atoms in the ^1^H NMR spectrum confirmed the *(E)-*configuration.

### Doses of 2HMC used in *in vitro* and *in vivo* assays

In the *Salmonella* mutagenicity test (Ames test), the doses of 2HMC used were 1, 10, 50, 100, 500, and 1000 μg/plate. These six doses used in our study cover a range from 1 to 1000 μg/plate in accordance to Mortelmans and Zeiger [22], which recommend a minimum of five doses and a range of at least three logs.

The doses of 25 and 50 mg/Kg of 2HMC used in the animal testing were based on previous studies in mice that used hydroxichalcones for biological activity evaluations [23, 24]. Dimethylsulfoxide (DMSO) was used to dissolve 2HMC and it was used as negative control in all performed assays.

### Ames test: *Salmonella* mutagenicity assay

#### Strains

*Salmonella typhimurium* tester strains TA98 (*hisD3052*) (*rfa*) (*uvrB*) (Amp^R^) and TA100 (*hisG46*) (*rfa*) (*uvrB*) (Amp^R^) [25] were kindly supplied by Dr. Bruce N. Ames, from the University of California (Berkeley, CA, United States).

#### Experimental procedure

The *S*. *typhimurium* histidine point mutation assay was followed [22]. A 0.1-mL aliquot of bacterial suspension (1–2 × 10^9^ cells/mL) of each strain (TA98 and TA100) was incubated with 1, 10, 50, 100, 500, and 1000 μg/plate 2HMC at 37°C for 25 min. A 2.0-mL aliquot of top agar [0.6% agar (lot no. 082613209, Kasvi, Curitiba, PR, Brazil), 0.5% NaCl, 50 μM L-histidine, and 50 μΜ biotin, at 45°C] was added to the test tubes and poured onto Petri dishes containing minimal agar medium [1.5% agar (lot no. 082613209, Kasvi, Curitiba, PR, Brazil), 2% glucose, and Vogel-Bonner medium E (MgSO_4_H_2_O, lot no. 1109865; C_6_H_8_O.7H_2_O, lot no. 1100795; K_2_HPO_2_, lot no. DCBB4100; Na_2_NH_2_PO_4_.H_2_O, lot no. 1208265, Vetec Química Fina Ltda., Duque de Caxias, RJ, Brazil)]. Each assay was performed three times in triplicate and included a negative control [20 μL dimethylsulfoxide (DMSO, lot no. 77320, Dinâmica Química Contemporânea Ltda., Diadema, SP, Brazil)] and a positive control [0.5 μg 4-nitroquinoline-1-oxide (4NQO, lot no. SLBD6960V, Sigma-Aldrich, São Paulo, SP, Brazil) per plate for TA98 and 3.0 μg sodium azide (lot no. 26628–22–8, Merck, Cotia, SP, Brazil) for TA100]. For the antimutagenicity evaluation, the same doses of 2HMC employed in the mutagenic evaluation were co-treated with their respective positive controls. After incubation at 37°C for 48 h, His^+^ revertant colonies were counted.

#### Statistical analysis

The software Sigma Stat 3.5 was used in all analyses. The results were expressed as mean ± standard deviation (SD). Mutagenicity results were evaluated using analysis of variance (ANOVA) and the Tukey’s test for differences among the means. Mutagenicity induction was measured by the mutagenic index (MI), calculated as the ratio between the number of colonies in the test treatment and the number of colonies in the negative control treatment. A substance is considered mutagenic when MI ≥ 2. In order to evaluate the antimutagenicity, the inhibition percentage of mutagenicity (IP) induced by the mutagen was calculated using the following formula [26]:

$$IP\;(\%)=\left[1-(\frac{TP-SR}{PCP-SR})\right]\;X\;100$$

where:

TP: number of His^+^ revertants on test plates (plates incubated with mutagen and test compound)

SR: spontaneous His^+^ revertants on negative control plates (tester strains incubated in the absence of test compound and mutagen)

PCP: number of His^+^ revertants on positive control plates (plates incubated with the mutagen alone)

#### Animal testing

This study was approved by the Animal Research Ethics Committee of the Federal University of Goiás (CEUA/UFG no. 013/14). Healthy, young male adult (8–12 weeks) outbred mice (*Mus musculus*, Swiss Webster), weighing 25–30 g, obtained from the animal facilities of the same university, were brought to the laboratory five days before the experiments and housed in plastic cages (40 cm × 30 cm × 16 cm), lined with wood shavings, kept at 24 ± 2°C and 55 ± 10% humidity, with a light-dark natural cycle of 12 h. Standard food pellets (appropriate commercial rodent diet Labina, Ecibra Ltda., Santo Amaro, SP, Brazil) and water were provided *ad libitum*.

#### Protocols *in vivo*

The animals were randomized into control and experimental groups, divided into 10 groups of five animals each group and weighed before the administration of the chemicals. All the doses of 2HMC were administered orally.

#### Control

The animals in Group 1 received 0.15 mL DMSO (negative control) administered orally, while the animals in Group 2 received 50 mg/kg (body weight) BW cyclophosphamide (CPA, lot no. 5L091, Baxter Hospitalar Ltda., São Paulo, SP, Brazil) (positive control) in a single intraperitoneal administration (ip).

#### Genotoxicity and cytotoxicity

The animals in Group 3 received a single dose of 50 mg/kg BW 2HMC (24 h), whereas those in Group 4 received a treatment only with 50 mg/kg BW 2HMC for five consecutive days (120 h).

#### Co-treatment

The animals in Groups 5 and 6 were respectively treated with 25 and 50 mg/kg BW 2HMC and concomitantly received a single dose of 50 mg/kg BW CPA ip each.

#### Pre-treatment

The animals in Groups 7 and 8 received 25 and 50 mg/kg BW 2HMC, respectively, for five consecutive days. On the last day, the animals in both groups received a single dose of 50 mg/kg BW CPA ip 2 h after the administration of 2HMC.

#### Post-treatment

The animals in Groups 9 and 10 were treated with a single dose of 50 mg/kg BW CPA ip. After 6 h and 12 h, the animals in Group 9 received 25 mg/kg BW 2HMC, whereas the animals in Group 10 received 50 mg/kg BW 2HMC.

All the animals were euthanized by cervical dislocation. The ones treated with CPA were euthanized 24 h after the administration, while those who received 2HMC alone were euthanized 24 h after the last administration of the chalcone. The bone marrow cells from both femurs of the animals were flushed using fetal calf serum (lot no. 0004, Cultilab Ltda., Campinas, SP, Brazil) and, after centrifugation (300× g, 5 min), the cells pellets were used for preparation of micronucleus test slides and the comet assay.

#### Micronucleus test

The micronucleus test was performed according to Von Ledebur and Schimid [27]. Bone marrow cells prepared as described above were smeared on glass slides, coded for blind analysis, air-dried, and fixed with absolute methanol (lot no. 1207433 COD: 000102.06, Vetec Química Fina Ltda., Duque de Caxias, RJ, Brazil) at room temperature for 5 min. The smears were stained with Giemsa (lot no. 13420C, New Prov Ltda., Pinhais, PR, Brazil), dibasic sodium phosphate (lot no. A0339828, Acros Organics, Morristown, NJ, United States), and monobasic sodium phosphate (lot no. 33791/11, Cromoline Química Fina, Diadema, SP, Brazil). In the micronucleus test we analyzed 2,000 polychromatic erythrocytes (PCE) per animal. Five animals were analyzed for each dose, thus a total of 10,000 PCE were analyzed per dose to determine the frequency of micronucleated polychromatic erythrocytes (MNPCE) using light microscopy (Olympus BH-2 10 × 100, Tokyo, Japan). Genotoxicity and antigenotoxicity were assessed by the frequency of MNPCE, whereas cytotoxicity was evaluated by the PCE and normochromatic erythrocytes (NCE) ratio (PCE/NCE), using a total of 10,000 cells for each dose.

#### Comet assay

The comet assay was performed using the alkaline method with few modifications [28]. Slides previously coated with normal melting point agarose (1.5%) received a mixture that contains 15 μl of bone marrow cells and 120 μl of low melting point agarose (0.75%) at 37°C. The mixture was spread on the slides with coverslips and taken to a cold chamber. After gelation, the coverslips were carefully removed. The slides were immersed in lysis solution protected from light [1% triton X-100 (lot no. DCBB3232, Vetec Química Fina Ltda., Duque de Caxias, RJ, Brazil), 10% DMSO, 2.5 M NaCl (lot no. 73516, Dinâmica Química Contemporânea Ltda., Diadema, SP, Brazil), 100 mM Na_2_EDTA (lot no. 2965C504, Invitrogen by Life Technologies, Itapevi, SP, Brazil), and 10 mM Tris (lot no. 0805008, Vetec Química Fina Ltda., Duque de Caxias, RJ, Brazil), pH 10.0] at 4°C for 12–24 h. Subsequently, the slides were incubated with freshly made alkaline solution [300 mM NaOH (lot no. 19804, Neon Comercial Ltda., São Paulo, SP, Brazil), 1 mM EDTA, pH > 13] at 4°C for 20 min for DNA unwinding. The electrophoresis was performed in the same buffer at 300 mA and 25 V/cm at 4°C for 30 min. After the electrophoresis, the slides were placed in a staining tray, covered with a neutralizing buffer (0.4 M Tris-HCl, pH 7.5), stained with ethidium bromide, and analyzed. For each treated group 500 nucleoids were analyzed,100 nucleoids per animal, using an epifluorescence Leica DM 2000 Citogen microscope (Leica Microsystems, Wetzlar, Germany), equipped with a Jenoptik ProgRes^®^ MF camera (Optronics, Goleta, CA, USA), driven by Lucia Cytogenetics^TM^ version 2.5 software (Laboratory Imaging Ltd, Prague, Czech Republic), 200 x magnification.

For the assessment of the genomic damage using the comet assay, the OpenComet^TM^ software, version 1.3 (Cometbio-OpenComet, Singapore) was employed. Using a novel and robust method for finding comets based on geometric shape attributes, this open-source software tool provides automated analysis of comet assay images segmenting the comet heads through image intensity profile analysis. As a result of automation, it is more accurate, less prone to human bias, and faster than manual analysis [29]. Since percentage of DNA in the tail (%DNA in tail) is commonly used in several studies for quantification of DNA damage [30–32], this parameter was also chosen in our study.

#### Statistical analysis for the micronucleus test and the comet assay

The genotoxic and antigenotoxic activities were evaluated using one-way ANOVA followed by the Tukey’s test, whereas cytotoxicity and anticytotoxicity were analyzed using the chi-square test. For the comet assay, the average and standard deviation of the %DNA in tail were considered for each treated group, and the one-way ANOVA test was used to compare the treated groups with their respective control groups. For genotoxicity and cytotoxicity evaluation, groups 3 and 4 were compared to the negative control (group 1). In the evaluation of antigenotoxicity, groups 5 and 6 (co-treatment), groups 7 and 8 (pre-treatment), groups 9 and 10 (post-treatment), were compared with the positive control (group 2), in order to evaluate 2HMC effects against CPA induced damage. The results were considered statistically significant when *p* < 0.05. The percentage reduction in CPA-induced genotoxicity was calculated using the following formula [33,34]:

$$\%\;Reduction=\left(\frac{A-B}{A-C}\right)\;X\;100$$

where:

A: MNPCE mean in CPA treatment (positive control)

B: MNPCE mean in antigenotoxic treatment (chalcone 2HMC + CPA)

C: MNPCE mean in negative control

## Results

### Ames test: *Salmonella* mutagenicity assay

The results of the mutagenic and antimutagenic evaluation, from three independent experiments carried out in triplicate, are presented in Table 1. The results obtained for the positive and negative controls are in agreement with Maron and Ames [25] and Mortelmans and Zeiger [22].

**Table 1:** Means ± standard deviation (SD) of histidine revertant colonies (obtained from three independent experiments carried out in triplicate), mutagenic index (MI), and inhibition percentage of mutagenicity (IP) for two tester strains of *Salmonella typhimurium*, TA98 and TA100, after treatment with different doses of the chalcone (*E*)-1-(2-hydroxyphenyl)-3-(4-methylphenyl)-prop-2-en-1-one) (2HMC).

Treatment	Mutagenicity				Antimutagenicity			
	TA 98		TA 100		TA 98		TA 100	
	Mean ± SD	MI	Mean ± SD	MI	Mean ± SD	IP (%)	Mean ± SD	IP (%)
Negative control^1^	20.66 ± 1.00^**A**^	1.00	143.00 ± 8.71^**A**^	1.00	21.99 ± 0.33	_	164.66 ± 38.08	_
Positive control^2^	509.55 ± 18.76	24.66	2157.66 ± 141.86	15.08	514.10 ± 80.52^**C**^	_	2088.33 ± 264.04^**C**^	_
2HMC 1 μg/plate	19.88 ± 3.02^**A**^	0.96	129.33 ± 14.64^**A**^	0.90	285.33 ± 64.45^**D**^	46.5	1549.00 ± 25.06^**D**^	28.0
2HMC 10 μg/plate	14.77 ± 1.07^**B**^	0.71	86.33 ± 8.96^**B**^	0.60	174.10 ± 10.80^**D**^	69.1	1508.33 ± 280.57^**D**^	30.2
2HMC 50 μg/plate	18.99 ± 0.87^**A**^	0.91	78.33 ± 12.22^**B**^	0.54	244.55 ± 56.07^**D**^	54.8	1453.00 ± 201.16^**D**^	33.1
2HMC 100 μg/plate	16.21 ± 0.51^**A**^	0.78	84.00 ± 6.08^**B**^	0.58	187.66 ± 27.56^**D**^	66.4	1530.66 ± 112.69^**D**^	29.0
2HMC 500 μg/plate	17.10 ± 2.26^**A**^	0.82	77.00 ± 7.55^**B**^	0.53	100.88 ± 6.44^**D**^	83.9	1443.00 ± 152.67^**D**^	33.6
2HMC 1000 μg/plate	15.33 ± 1.33^**B**^	0.74	68.00 ± 9.64^**B**^	0.47	64.99 ± 0.87^**D**^	91.3	1445.33 ± 98.33^**D**^	33.5

^1^Negative control: 20 μL dimethylsulfoxide (DMSO).

^2^Positive control: 0.5 μg 4-nitroquinoline-1-oxide (4NQO) per plate for TA98 and 3.0 μg of sodium azide for TA100.

All values are means ± SD of three independent experiments.

Statistical analysis: one-way ANOVA and the Tukey’s test. Mutagenicity:

^**A**^No significant difference in comparison with the negative control (*p* > 0.05).

^**B**^Significant difference in comparison with the negative control (*p* < 0.05). Antimutagenicity:

^**C**^No significant difference in comparison with the positive control (*p* > 0.05).

^**D**^Significant difference in comparison with the positive control (*p* < 0.05).

In the evaluation of mutagenicity, the doses of 2HMC tested (1, 10, 50, 100, 500, and 1000 μg/plate) did not cause a significant increase in the number of His^+^ revertant colonies in either tester strains, TA98 or TA100 (*p* > 0.05). Furthermore, none of the strains tested reached MI ≥ 2 or dose-response effect with 2HMC treatment, with the highest induction occurring for strain TA98, which reached MI = 0.96 at the dose of 1 mg/plate.

The results of the Ames test demonstrated that 2HMC did not exhibit a mutagenic effect. Tester strains TA98 and TA100 exhibited MI lower than the negative control (MI = 1.00) at all doses, indicating that this chalcone possibly had a mild cytotoxic effect against *S*. *typhimurium* cells. Based on the results of the antimutagenic evaluation, at all doses tested (1, 10, 50, 100, 500, and 1000 μg/plate) 2HMC showed a significant decrease in the number of His^+^ revertant colonies in tester strains TA98 and TA100 compared to the respective positive controls (*p* < 0.05). At doses of 1, 10, 50, 100, 500, and 1000 μg/plate, the results of IP were 46.5%, 69.1%, 54.8%, 66.4%, 83.9%, and 91.3% for strain TA98, and 28.0%, 30.2%, 33.1%, 29.0%, 33.6%, and 33.5% for strain TA100, respectively. Therefore, 2HMC exhibited significant antimutagenic action at all doses, mainly in tester strain TA98, with IP 91.3% at the dose of 1000 μg/plate. Hence, it is possible to affirm that at all doses tested 2HMC was able to significantly protect DNA from the mutagens sodium azide and 4NQO.

### Micronucleus test

The frequencies of MNPCE and PCE/NCE ratio of groups co-treated, pre-treated, or post-treated with 2HMC and CPA are presented in Table 2. The negative control group (Group 1) had low MNPCE values, as expected, and the positive control group (Group 2) exhibited a significant increase in MNPCE compared to the negative control group (*p* < 0.05), confirming the sensitivity of the test.

**Table 2:** Effects of treatments with different doses of the chalcone (*E*)-1-(2-hydroxyphenyl)-3-(4-methylphenyl)-prop-2-en-1-one) (2HMC) on the frequency of micronucleated polychromatic erythrocytes (MNPCE) and polychromatic/normochromatic erythrocyte ratio (PCE/NCE) in bone marrow cells of mice.

Group	Treatment	MNPCE/2000 PCE	MNPCE reducion (%)	PCE/NCE
**Control**				
1	Negative control (DMSO)^1^	4.2 ± 0.45		1.1 ± 0.05
2	Positive control (CPA)^2^	27.2 ± 1.3		0.71 ± 0.02
**Genotoxicity and Cytotoxicity**				
3	2HMC 50 mg/kg BW (24 h)	4.8 ± 0.84		0.87 ± 0.04 ^**§**^
4	2HMC 50 mg/kg BW (120 h)	5.2 ± 1.30		0.77 ± 0.1 ^**§**^
**Co-treatment**				
5	2HMC 25 mg/kg BW (24 h) + CPA	17 ± 1.58^*^	44.3	0.78 ± 0.02
6	2HMC 50 mg/kg BW (24 h) + CPA	15.8 ± 3.03^*^	49.6	0.71 ± 0.02
**Pre-treatment**				
7	2HMC 25 mg/kg BW (120 h) + CPA	13.2 ± 2.16^*^	60.9	0.63 ± 0.02
8	2HMC 50 mg/kg BW (120 h) + CPA	10.8 ± 1.92^*^	71.3	0.53 ± 0.02 ^*^
**Post-treatment**				
9	CPA + 2HMC 25 mg/kg BW	27.4 ± 1.81		0.71 ± 0.01
10	CPA + 2HMC 50 mg/kg BW	26.8 ± 0.84		0.70 ± 0.01

^1^Negative control: 0.15 mL dimethylsulfoxide (DMSO) administered orally.

^2^Positive control: 50 mg/kg BW cyclophosphamide (CPA) diluted in physiological saline and administered ip.

Groups 3 and 4, treated only with 2HMC, were compared to the negative control group.

Groups 5, 6, 7, 8, 9, and 10, co-, pre-, or post-treated were compared to the positive control group.

* Significant compared to the positive control (*p* < 0.05).

^**§**^Significant compared to the negative control (*p* < 0.05)

The data were analyzed using one-way ANOVA, Tukey’s test, and chi-square test.

Regarding the genotoxicity, MNPCE frequency in Group 3, which received only 50 mg/kg BW 2HMC (24 h), was 4.8 (per 2000 PCE), whereas in the negative control group it was 4.2. Given that, this dose of 2HMC did not cause a significant increase in MNPCE frequency at 24 h compared to the negative control group (*p* > 0.05), thus the absence of genotoxic effects in 24 h of exposure to this chalcone was proven. Group 4, which received only 50 mg/kg BW 2HMC for five consecutive days (120 h), did not have a significant increase in MNPCE frequency (5.2) compared to the negative control group (4.2) (*p* > 0.05), demonstrating absence of genotoxic effect even after 120 h of exposure.

The PCE/NCE ratio is used as an indicator of cytotoxicity. In Group 3, treated only with 50 mg/kg BW 2HMC (24 h), the PCE/NCE ratio was 0.87, while in the negative control group it was 1.1. These results demonstrate that this treatment with 2HMC caused a significant decrease in the PCE/NCE ratio compared to the negative control group (*p* < 0.05), suggesting a mild cytotoxic effect of the chalcone at this dose and 24 h of exposure. Group 4 showed significant decrease in PCE/NCE ratio (0.77) compared to the negative control group (1.1) (*p* < 0.05), which also suggests a moderate cytotoxic effect of 2HMC at this dose and 120 h of exposure.

#### Co-treatment

In the evaluation of 2HMC antigenotoxicity, MNPCE frequencies in Groups 5 and 6, treated with 25 and 50 mg/kg BW, were 17 and 15.8, respectively, whereas in the positive control group it was 27.2. At both doses, 2HMC caused a significant decrease in MNPCE frequencies compared to the positive control group (*p* < 0.05). Based on these results, both doses of 2HMC reduced CPA-induced genotoxicity, by 44.3% and 49.6%, respectively, indicating its antigenotoxic effect.

Regarding to anticytotoxicity assessed in the co-treatment, Groups 5 and 6, treated with 25 and 50 mg/kg BW 2HMC, had PCE/NCE ratios of 0.78 and 0.71, respectively, while in the positive control group it was 0.71. According to these results, neither of the treatments with 2HMC cause a significant increase in PCE/NCE ratio compared to the positive control group (*p* > 0.05), evidencing absence of anticytotoxic effect.

#### Pre-treatment

The evaluation of 2HMC antigenotoxicity showed that MNPCE frequencies in Groups 7 and 8, which received 25 and 50 mg/kg BW 2HMC, were 13.2 and 10.8, respectively. These results are significantly lower than those obtained for the positive control group (27.2) (*p* < 0.05). Therefore, both doses of 2HMC reduced the genotoxic activity of CPA, by 60.9% and 71.3%, respectively, revealing a strong antigenotoxic effect of the 2HMC in pre-treatment.

For the anticytotoxic assessment in pre-treatment the PCE/NCE ratios in Groups 7 and 8 were 0.63 and 0.53 respectively, whereas in the positive control group was 0.71. At these doses, no increase was observed in PCE/NCE ratio compared to the positive control group, demonstrating the 2HMC absence of anticytotoxic effect. Unlike that, it was observed that 50 mg/kg BW 2HMC dose significantly reduced the PCE/NCE ratio compared to the positive control group (*p* < 0.05). Thus, 2HMC enhanced the cytotoxic action of CPA in pre-treatment.

#### Post-treatment

Groups 9 and 10, treated with 25 and 50 mg/kg BW 2HMC, had MNPCE frequencies of 27.4 and 26.8 and PCE/NCE ratios of 0.71 and 0.70, respectively, not presenting a significant difference compared to the positive control group (*p* > 0.05). According to these results, none of the doses of 2HMC exhibited antigenotoxic or anticytotoxic effects in post-treatment.

### Comet assay

**Fig 2:**
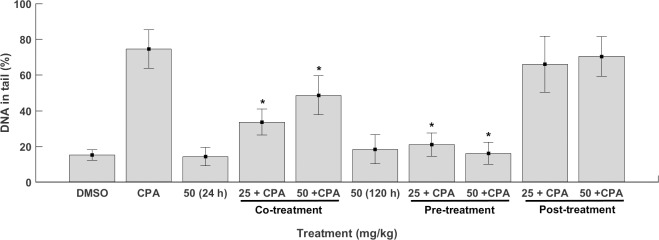
Assessment of the genotoxic and antigenotoxic activities of the chalcone (*E*)-1-(2-hydroxyphenyl)-3-(4-methylphenyl)-prop-2-en-1-one) (2HMC) at different doses in mice bone marrow cells using the comet assay estimated by the parameter %DNA in tail. DMSO, dimethylsulfoxide (negative control); CPA, cyclophosphamide (50 mg/kg BW) (positive control). ANOVA and the Tukey’s test: *significant compared to the positive control (*p* < 0.05). Groups 3 and 4, treated only with 2HMC, were compared to the negative control. Groups 5, 6, 7, 8, 9, and 10, co-, pre-, or post-treated were compared to the positive control.

Bone marrow cells from all the groups of mice treated were used to evaluate DNA damage using the comet assay. The measurement of the parameter %DNA in tail is presented in Fig 2.

Examining the results of the comet assay, 2HMC did not induce significant DNA damage at the dose of 50 mg/kg BW at 24 h and 120 h. This dose did not cause significant difference in the parameter %DNA in tail compared to the negative control group (*p* > 0.05) (Fig 2), demonstrating absence of genotoxic effects at 24 h and 120 h.

Also, Fig 2 shows the extent of DNA damage in mice bone marrow cells simultaneously exposed to 2HMC and CPA (co-treatment). The two doses tested, 25 and 50 mg/kg BW, promoted a significant reduction in the extent of DNA damage compared to Group 2 (positive control), treated with CPA alone (*p* < 0.05), exhibiting antigenotoxic effect in the co-treatment.

Mice bone marrow cells exposed to both doses of 2HMC for five consecutive days (120 h) followed by CPA showed the highest reductions of DNA damage compared to the positive control group (*p* < 0.05). Based on these results, it is possible to affirm that 2HMC clearly protected DNA against CPA-induced damage.

The post-treatment with 25 and 50 mg/kg BW 2HMC did not statistically differ from the CPA positive control group regarding %DNA in tail. These results show that neither of the doses of 2HMC tested decreased DNA damage in post-treatment.

## Discussion

The aim of the present study was to evaluate the genotoxic, cytotoxic, and protective effects of 2HMC using *in vitro* and *in vivo* assays. The use of the *in vitro* mutagenicity test (Ames test) combined with the *in vivo* mouse bone marrow micronucleus test has been recommended in several international guidelines to test the genotoxic/antigenotoxic potential of various types of substances [35]. In addition to these tests, the comet assay also has been used to assess DNA damage [36,37].

In our study, 2HMC did not exhibit mutagenic effect by Ames test, since none of the doses reached MI ≥ 2 on either tester strains (Table 1). Several studies have reported the lack of mutagenic action for many classes of chalconas [38,39]. Furthemore, in a study using diferent hydroxychalcones, 4’5’-dimethyl-2’,3,4-trihydroxylchalcone, 6 methyl-2’,3,4-trihydroxychalcone, and 3,3’,4’,7-tetrahydroxyflavone, none of them presented mutagenic effect by the Ames test [40]. Also in our study, all the doses of 2HMC exhibited MI lower than the negative control (MI = 1.00) in both tester strains, indicating a possible toxic effect of this chalcone in *S*. *typhimurium*. This mild toxicity is in accordance with an early study using 2HMC, which presented moderate antibacterial effect against *Bacillus megaterium* and *Klebsiella* sp. [41].

The mutagenic agents 4NQO and sodium azide were employed in the present study to evaluate the antimutagenic effect of 2HMC. 4NQO induces intracellular oxidative stress, generating reactive oxygen species (ROS) and metabolic products that can bind to DNA, mainly to guanine residues, causing mutations [42]. Sodium azide produce reactive metabolites and induces ROS generation that can bind to DNA, causing point mutations [43,44].

In the antimutagenicity evaluation using the Ames test, 2HMC exhibited significant antimutagenic activity against both mutagens (4NQO and sodium azide) at all doses tested. Tester strain TA98 showed the higher antimutagenic effect, reaching an IP of 91.3% against mutation induced by 4NQO and protected DNA mainly against frameshift mutations (Table 1). This significant antimutagenicity of 2HMC in our study could be related to the presence of an *α*,*β*-unsaturation in its structure, which creates a nucleophilic center at *β*-carbon [45], interacting with 4NQO and sodium azide metabolites, reducing their mutagenic effects.

In addition to the Ames mutagenicity test, the micronucleus test and the comet assay in mice were performed in our study, since both tests display different mechanisms of DNA damage [46–48]. In the present study, neither the administration of 2HMC alone at 24 h nor its usage for 120 h exhibited genotoxic effects using the micronucleus test (Table 2) and comet assay (Fig 2). These results are similar to recent findings involving two main chalcones, 4-hydroxyderricin and xanthoangelol, which did not display genotoxic effects in bone marrow erythrocytes of mice using the chromosome aberration assay and the *in vivo* micronucleus test [49].

Several authors have reported that structural, geometrical and physico-chemical patterns of several compounds are essentially related to their biological activities [50]. Previous studies of Density Functional Theory (DFT) and ^1^H NMR shifts demonstrated that 2HMC exists preferably in the *trans*-conformation, which is most thermodynamically stable [51,52]. This *trans*-conformation allows a hydrogen bond between the C = O and the 2’-OH, in accordance with the crystallographic characterization of 2HMC, thus facilitating the electron transfer mechanism [53].

Donating electrons, these compounds generate aryloxyl (ArO^.^) radicals that could interact with free radicals, exerting an antioxidant effect [54]. Studies have attributed DNA damage preventive activity to ROS scavenging capacity [55]. The antigenotoxicity evaluation showed that co-treatment and pre-treatment with 2HMC were able to significantly reduce the frequency of micronucleus in the micronucleus test and decreased the number of DNA breaks in the comet assay.

CPA is rapidly absorbed by the organism and converted by P450-enzymes into active metabolites, resulting in increased ROS generation and lipid peroxidation [56,57]. These ROS induce DNA damage by promoting DNA cross-links and breaks, which are crucial in genotoxic and carcinogenic process [58–60]. In recent studies, 2HMC revealed antioxidant activity against superoxides and it was also able to inhibit lipid peroxidation [61–63]. Structure-activity relationship studies showed that ArO^.^ radical formed in the electron transfer mechanism of 2’-hydroxychalcones, binds to ROS, deactivating these groups and displaying an antioxidant activity [54]. Also, a study using *in vitro* DPPH assay reported that 2HMC presented 10-fold greater free radical scavenging activity when compared to other B ring non-substituted 2’-hydroxychalcones [63–65]. Therefore, the antimutagenic activity of 2HMC observed in the micronucleus test and comet assay could be partially attributed to its high free radical scavenging capacity, which protected DNA from the harmful actions of CP metabolites.

From all tested groups, the pre-treatment was the most effective in reducing the frequency of MNPCE and DNA breaks, indicating a greater chemopreventive action of 2HMC against CPA damaging effects. However, animals post-treated with 2HMC did not present a significant reduction of MNPCE and DNA breaks, revealing that this compound was not able to induce DNA repair system.

It’s important emphasize that the ArO^.^ radical displays a significant role in the ROS scavenging mechanisms, but when local concentrations of ArO^.^ increase, it also participates in pro-oxidant reactions [54]. This dual characterist of ArO^.^ radical could explain the moderate cytotoxic effect shown at groups treated only with 2HMC (groups 3 and 4), mainly at 120 h treatment. The ability of polyphenols to generate free radical species has been reported to interfer the mitochondrial membrane potential, thus inducing apoptosis [66].

In our results, animals pre-treated with 2HMC (groups 7 and 8) presented a decrease in the PCE/NCE ratio compared to the positive control, revealing a synergistic action of 2HMC and CPA. However, even 2HMC presenting a cytotoxic effect at pre-treatment, this treatment presented the lowest MNPCE frequencies. This fact may indicate that these cells might undergo an apoptosis process, which could have reduced the genotoxicity promoted by CPA.

Therefore, 2HMC showed antimutagenic effect by Ames test and antigenotoxic activity in the micronucleus test and comet assay. The capacity of protecting DNA against damage induced by different mutagens indicates that 2HMC presents an interesting profile for the development of chemopreventive drugs.

## Conclusion

The results obtained in this study using the Ames test, micronucleus test, and comet assay showed that 2HMC did not exhibit mutagenic and genotoxic effects. 2HMC presented a moderate cytotoxicity in mouse bone marrow cells in micronucleus test, mainly at pre-treatment. Regarding the protective effects of 2HMC, this chalcone reduced DNA damage promoted by genotoxic agents sodium azide, 4NQO, and CPA (in co-treatment and pre-treatment). The absence of effect in post-treatment indicated that this compound did not act on DNA repair processes. We suggested that protective activity of 2HMC found in the present study could be due to an antioxidant activity and its capacity to form complexes with molecules, preventing DNA damage. Therefore, this protective effect against different mutagens suggests that 2HMC could be a probable candidate for the development of chemopreventive agents.
